# The evaluation of synchronous and asynchronous online learning: student experience, learning outcomes, and cognitive load

**DOI:** 10.1186/s12909-024-05311-7

**Published:** 2024-03-22

**Authors:** Chih-Tsung Hung, Shou-En Wu, Yi-Hsien Chen, Chen-Yeu Soong, Chien‑Ping Chiang, Wei‑Ming Wang

**Affiliations:** 1 Department of Dermatology, Tri-Service General Hospital, National Defense Medical Center, No.325, Sec. 2, Chenggong Rd., Neihu Dist., 114 Taipei, Taiwan; 2 Graduate Institute of Medical Sciences, National Defense Medical Center, Taipei, Taiwan; 3 Department of Biochemistry, National Defense Medical Center, Taipei, Taiwan

**Keywords:** Online learning, Cognitive load, Self-efficacy, Student satisfaction, Medical education

## Abstract

**Background:**

The abrupt onset of the COVID-19 pandemic compelled universities to swiftly establish online teaching and learning environments that were not only immediately deployable but also conducive to high-quality education. This study aimed to compare the effectiveness of the online synchronous and asynchronous teaching formats in the dermatology lecture for undergraduate medical students, including academic performance, self-efficacy, and cognitive load.

**Methods:**

A total of 170 fourth-year undergraduate medical students attending the dermatology lecture were included. The lecture was delivered using both the synchronous method (live online lecture via Webex meeting) and the asynchronous method (lecture videos shared on YouTube). The students had the freedom to choose their preferred method of attending the online lecture. The study assessed three main aspects: (1) learning outcomes measured through pretest, posttest, and retention test scores; (2) cognitive load experienced by students, including mental load and mental effort measured using eight items; and (3) satisfaction levels with each online teaching format.

**Results:**

In this study, 70 students opted for the synchronous online lecture, while 100 students chose the asynchronous online lecture. Both synchronous and asynchronous teaching methods exhibited significant improvements in post and retention test scores compared to the pretest. Satisfaction levels, rated on a scale of 0–5, were generally high for both teaching methods, with no significant differences observed (4.6 for synchronous, 4.53 for asynchronous; *p* =.350). Regarding cognitive load, the synchronous method showed a significantly lower level than the asynchronous method (*p* =.0001). Subgroup analysis revealed no difference in mental effort (*p* =.0662), but the level of mental load was lower in the synchronous method (*p* =.0005).

**Conclusions:**

Both synchronous and asynchronous online teaching methods demonstrated improvements in learning outcomes and high levels of student satisfaction. However, the cognitive load experienced by students was lower in the synchronous setting compared to the asynchronous setting. These findings remind health professions educators that they would consider the students’ cognitive load when designing online curricula.

**Supplementary Information:**

The online version contains supplementary material available at 10.1186/s12909-024-05311-7.

## Background

The global spread of COVID-19 has expanded a distinctive moment for the progress of online education. With students originating from 190 countries, numbering in the billions, have been required to transition to remote learning, termed emergency remote teaching (ERT) [1–3]. When considering the temporal dimension, online learning can be broadly classified into two categories: asynchronous and synchronous [4]. Asynchronous online learning allows students to independently access online curricular materials at their convenience, freeing them from temporal and spatial constraints imposed by teacher-student interaction [5]. While providing flexibility in terms of time, asynchronous learning places a higher demand on students’ self-discipline due to limited interaction with instructors. In contrast, synchronous online learning requires students and teachers to synchronize schedules for real-time communication, simulating a physically present classroom environment despite geographic separation [5]. Hence, according to some scholars, asynchronous online learning is considered “individually based,” while synchronous online learning is perceived as resembling traditional classroom instruction [6].

Undoubtedly, online education brings numerous advantages, including convenience, enhanced interaction, and improved learning effectiveness. However, it is important to acknowledge reported disadvantages, such as technical challenges, subpar academic performance, and limited practical knowledge acquisition [7, 8]. Technical challenges may include the absence of personal computers or reliable internet connections, particularly for individuals from lower socioeconomic backgrounds. To assess whether the benefits outweigh the drawbacks, evaluating satisfaction levels is considered a crucial indicator of online educational program quality [9]. 

Approximately four decades ago, Albert Bandura introduced the concept of “self-efficacy” [10]. Self-efficacy refers to the belief in one’s ability to effectively organize and execute actions necessary to achieve specific goals [11]. It significantly impacts various performance aspects crucial for learning, including effort exertion and persistence in completing tasks [12]. In online learning, self-efficacy is recognized as a critical factor influencing learners’ performance and persistence. It serves as a reliable predictor of academic achievements and contributes to adaptability, perseverance, and effective coping, even in the face of limited prior online learning experience [13].

In the design of effective medical education, it is essential to consider the concept of cognitive load (CL) [14]. CL encompasses an individual’s cognitive capacity used for task performance, learning, or problem-solving [15, 16]. It consists of both a causal dimension involving the interaction between individual and task characteristics, and an assessment dimension including quantifiable elements like mental load (ML), mental effort (ME), and performance [15]. ML represents task-related cognitive capacity, while ME reflects an individual’s cognitive capacity during task engagement. According to Sweller et al., ML and ME are distinct constructs usually positively correlated [17]. Performance can be considered one aspect of CL, or in some cases, an indicator of CL [15, 18]. In this study, we investigated students’ ML, ME, and performance to comprehensively understand all aspects of students’ CL.

Research findings on the impact of synchronous and asynchronous teaching settings on student performance exhibit some degree of ambiguity. Nieuwoudt JE (2020) discovered that there was no difference in student achievement based on whether students attended synchronous virtual classes or viewed recordings of these classes [19]. However, the actual time students spent participating in and engaging with the online learning system did significantly influence their academic success. The study aims to evaluate the acceptance of synchronous and asynchronous online learning environments among fourth-year undergraduate medical students, exploring academic performance, self-efficacy, and cognitive load.

## Materials and methods

### Ethics

The Institutional Review Board of Tri-Service General Hospital conducted a thorough review and granted approval for this study (TSGHIRB No.: C202105012).

### Research design, setting, and sample

In Taiwan, the medical education system encompasses a six-year curriculum. The initial two years focus on liberal education, followed by a three-year preclinical stage where students explore the intricacies of both healthy and diseased bodily functions. The subsequent clinical stage, the sixth year, involves a transition from the classroom to the hospital setting. During this phase, students actively engage in hands-on learning through direct patient care, actively participating in medical procedures and patient management under the guidance of residents and attending physicians. This active involvement positions them as integral members of the healthcare team.

Specifically, in the fourth year, all medical students attend lectures of the cutaneous system, which comprises 18 different sessions. Our study was done in a lecture on dermatitis covers topics such as atopic dermatitis, nummular eczema, lichen simplex chronicus, prurigo nodularis, contact dermatitis, seborrheic dermatitis, and asteatotic dermatitis. The delivery of this lecture incorporates both synchronous methods, utilizing online live lectures via Webex meetings, and asynchronous methods, featuring lecture videos shared on YouTube. Students had the autonomy to choose their preferred method of attending the online lecture. Out of the initial group of 175 students, 5 had incomplete data and were consequently excluded from the analysis. This exclusion resulted in a final sample size of 170 out of 175, representing 97.14% of the initial cohort.

The synchronous module consisted of live lectures conducted using Cisco Webex Meetings, an online meeting app, at a scheduled date and time. Both the instructor and students participated in the online lecture through the app. This method allowed students to engage in discussions and address their questions either during the lecture or immediately afterward.

In the asynchronous module, students were provided with the video link, pretest, posttest, and questionnaire through the student response system (SRS) called Zuvio APP. Zuvio APP is an online platform that facilitates interaction and feedback. Within this app, students had the opportunity to ask questions, and the teaching faculty promptly responded to them [20].

Each class within the study incorporated pretest, posttest, and retention test assessments consisting of five different questions. The test quizzes underwent review by six dermatologists. The lecture commenced with the administration of the pretest, followed by the posttest immediately after the lectures. One week later, the short-term retention test was administered. The quizzes in the pretest and posttest were the same, but the quizzes in retention test were different. All three scores (pre-test, post-test, and retention test) were deemed valid and utilized for statistical analysis.

The questionnaire pertaining to self-efficacy for learning and performance was administered both before and after the lectures, employing a quantitative 5-point Likert-type response scale ranging from 1 (strongly disagree, representing the lowest degree) to 5 (strongly agree, representing the highest degree; Table S1-S4) [21]. The questionnaire gauging students’ cognitive load was administered after the lectures utilizing a 7-point Likert-type response scale, ranging from 1 (strongly disagree, the lowest degree) to 7 (strongly agree, the highest degree; Table S5) [22]. It includes measures of students’ ML and ME as control variables in educational research. Additionally, a satisfaction questionnaire, using a 5-point Likert-type response scale ranging from 1 (strongly disagree, the lowest degree) to 5 (strongly agree, the highest degree), was administered after the lectures. The satisfaction questionnaire aimed to evaluate students’ experiences with each online teaching module.

This study encompassed questionnaires to explore students’ opinions on self-efficacy for learning and performance, cognitive load, and their preference for the online teaching module. To ensure anonymity and promote honest responses, the survey did not collect any student identifiers, allowing participants to express their opinions without fear of recognition.

The mean ± 95% confidence intervals (CIs) were used to express the scores of the pretest, posttest, and retention test. The differences within each module were analyzed using the Wilcoxon matched-pairs signed rank test. To compare the two modules, Mann-Whitney U-test was employed. Statistical significance was determined with a two-tailed *p*-value threshold of less than 0.05.

## Results

**Fig. 1:**
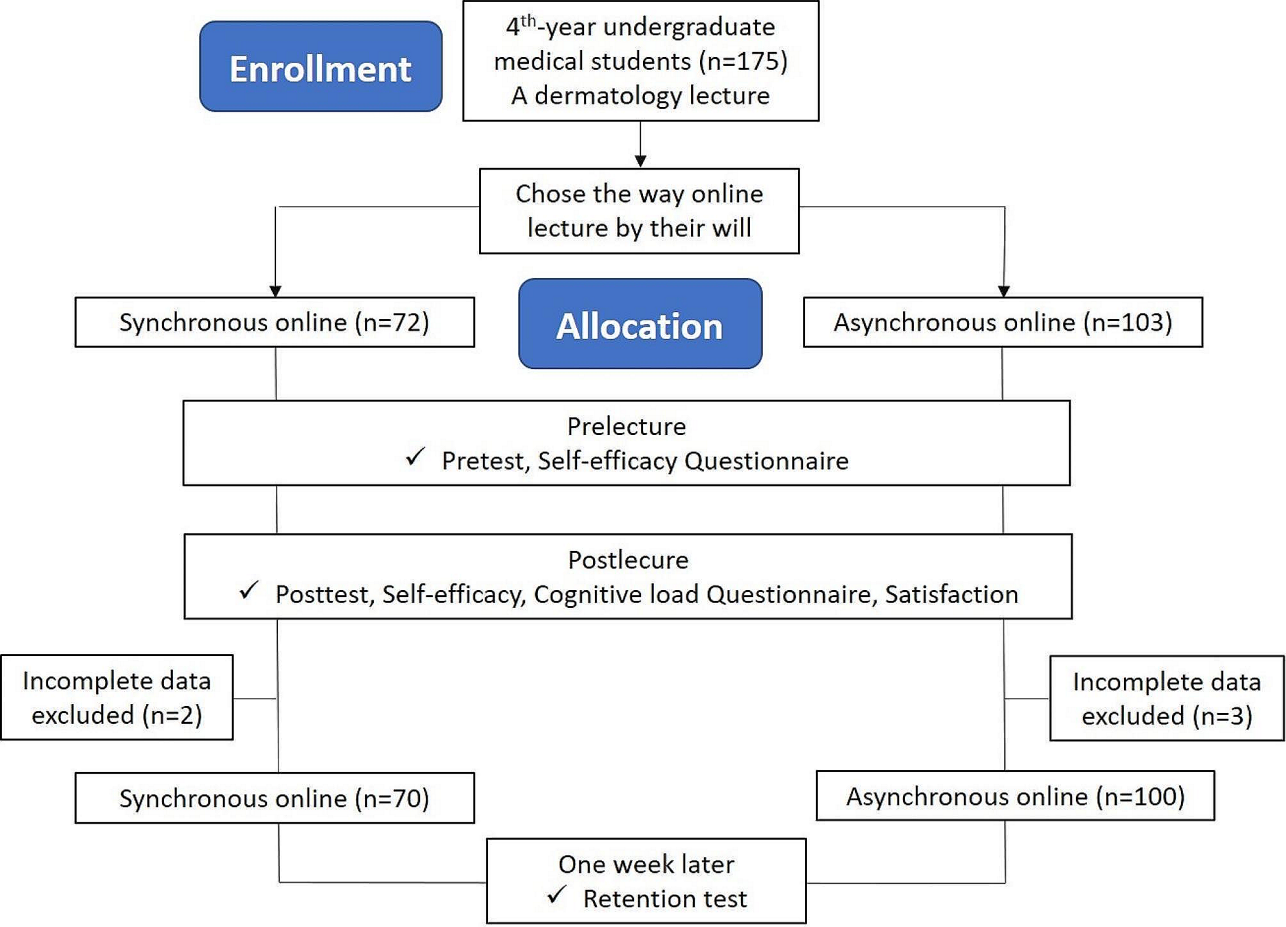
The flowchart of study population selection

A total of 175 4th -year medical students were enrolled in the preclinical dermatologic lecture at National Defense Medical Center, and they can choose the way to attend the lecture, either synchronized or unsynchronized online methods. Of these students, there were 72 students chose the synchronous module, and 103 students chose the asynchronous module. However, there were 5 students with incomplete data and were excluded from the analysis (2 students in the synchronous module; 3 students in asynchronous module, Fig. 1). In each group, they had to complete pretest, posttest, retention test, and questionnaire.

### Learning outcomes within synchronous and asynchronous online teaching

**Fig. 2:**
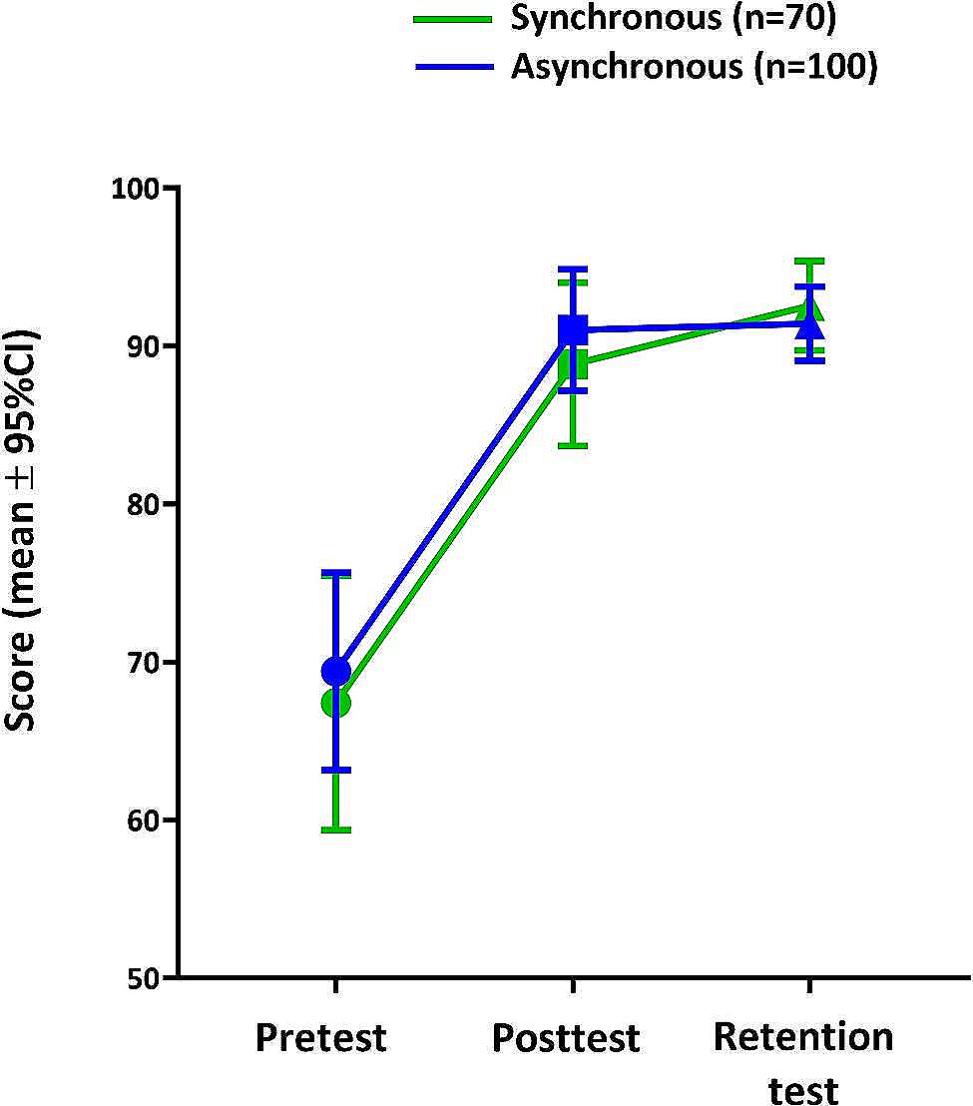
Mean scores of pre, post, and retention tests in synchronous and asynchronous modules

By comparing the scores of the posttest and retention test with those of the pretest, we evaluated the learning outcome improvements for each teaching module. The analysis yielded statistically significant improvements in both the posttest and retention scores for both instructional methods (*p* <.0001, as shown in Table 1; Fig. 2).

**Table 1:** Mean scores of pre, post, and retention tests in synchronous and asynchronous modules

Online module	Pretest score Mean (95% CI)	Posttest score Mean (95% CI)	Post vs. Pre-test		Retention Test Mean (95% CI)	Retention vs. Pre-test	
			Cohen’s d	*p* value		Cohen’s d	*p* value
Synchronous (*n* = 70)	67.43 (7.90)	88.86 (5.08)	0.755	< 0.0001	92.57 (2.78)	0.994	< 0.0001
Asynchronous (*n* = 100)	69.4 (6.17)	91 (3.79)	0.827	< 0.0001	91.4 (2.31)	0.926	< 0.0001

### Differences in learning outcomes between synchronous and asynchronous online teaching

To determine any difference in learning performance between the asynchronous and synchronous methods, we compared the scores of the pretest, posttest, and retention test across the two groups. The findings indicated no significant difference between the asynchronous and synchronous methods (pretest, *p* =.7785; posttest, *p* =.5559; retention test, *p* =.4435; Table 2).

**Table 2:** Comparison of pre, post, and retention test scores between synchronous and asynchronous modules

	Test score, Mean (95% CI)	Cohen’s d	*p* value
**Pretest**			
Synchronous	67.43 (7.90)	0.06	0.7785
Asynchronous	69.4 (6.17)		
**Posttest**			
Synchronous	88.86 (5.08)	0.103	0.5559
Asynchronous	91 (3.79)		
**Retention test**			
Synchronous	92.57 (2.78)	0.099	0.4435
Asynchronous	91.4 (2.31)		

### Student response to self-efficacy for learning and performance within synchronous and asynchronous online teaching

To ascertain the impact of synchronous and asynchronous online teaching on students’ self-efficacy for learning and performance, we compared the individual differences between pre-lecture and post-lecture self-efficacy scores. The results revealed a significant improvement in self-efficacy for both instructional methods (*p* <.0001, Table 3, Table S1, and Table S2).

**Table 3:** Comparison of self-efficacy for learning and performance in synchronous and asynchronous modules

Self-Efficacy Online module	Pre-lecture, Mean	Post-lecture, Mean	Post vs. Pre *p* value
Synchronous	3.91	4.29	< 0.0001
Asynchronous	3.88	4.26	< 0.0001
Synchronous vs. Asynchronous,			
Cohen’s *d*	0.03	0.036	-
*p* value	0.5004	0.6388	-

To determine any disparity in self-efficacy for learning and performance between the asynchronous and synchronous methods, we compared the scores of the questionnaire across the two groups. The results indicated no significant difference between the synchronous and asynchronous methods (pre-lecture, *p* =.5004; post-lecture, *p* =.6388; Table 3, Table S3, and Table S4).

### Differences in student response to cognitive load between synchronous and asynchronous online teaching

We aimed to examine the impact of synchronous and asynchronous online teaching on students’ cognitive load (Table 4 and Table S5). The results indicated that students’ cognitive load was significantly lower in the synchronous online teaching compared to the asynchronous online method (2.53 vs. 2.84, *p* =.0001, as shown in Table 4). However, the effect sizes were small. In subgroup analysis, the ML was significantly lower in the synchronous method compared to the asynchronous method (2.52 vs. 2.86, *p* =.0005). Although the ME was lower in the synchronous method than in the asynchronous method, the difference was not statistically significant (2.548 vs. 2.79, *p* =.0662).

**Table 4:** Comparison of cognitive load between synchronous and asynchronous modules

Online module	Synchronous	Asynchronous	Cohen’s d	*p* value
Cognitive load	2.53	2.84	0.218	0.0001
Mental load	2.52	2.86	0.244	0.0005
Mental effort	2.548	2.79	0.173	0.0662

### Students’ satisfaction with synchronous and asynchronous online teaching

To assess students’ satisfaction with synchronous and asynchronous online teaching, we administered a questionnaire using a Likert 5-point scale (Table 5). There was no statistically significant difference between the synchronous module and the asynchronous module [Mean score (SD): 4.6 (0.55) for synchronous, 4.53 (0.54) for asynchronous, respectively, *p* =.350].

**Table 5:** Comparison of students’ satisfaction between synchronous and asynchronous modules

Online module	Satisfaction, Mean (SD)	Cohen’s d	*p* value
Synchronous	4.6 (0.55)	0.129	0.350
Asynchronous	4.53 (0.54)		

## Discussion

The objective of this study was to assess the students’ academic performance, self-efficacy, and cognitive load in two online learning methods. The results of our study revealed that both methods significantly improved students’ learning. When comparing the two online methods, the synchronous method showed a trend toward higher scores than the asynchronous method but did not reach statistical significance. These findings align with a previous meta-analysis that also found no significant difference in students’ performance scores when comparing synchronous and asynchronous education methods [23]. Similar conclusions were drawn in a study by Nieuwoudt JE at Southern Cross University in Australia, which found no statistically significant difference in final grades between attending synchronous virtual classes and watching recorded classes [19].

Self-efficacy was examined to know the different online modules influenced students’ ability to accomplish a task and confidence to perform that task [24]. Self-efficacy is an important predictor of academic success, as an increase in self-efficacy promotes one’s engagement in learning and improves learning outcomes [25]. Compared to students’ responses to self-efficacy at pre-lecture, both methods showed significant improvement at post-lecture (*p* <.0001). There is a trend toward higher scores in the synchronous online method than in the asynchronous method, but that this did not reach statistical significance. This suggests that both online education methods are effective in improving students’ self-efficacy. Previous studies revealed that students who possessed higher levels of self-efficacy demonstrated a greater likelihood of actively participating in the online course and persevering throughout the semester [26, 27]. It also aligns with the theoretical concept of learning presence, which emphasizes the significance of students’ confidence in their ability to succeed as a crucial factor in their engagement with online coursework [28].

The cognitive load model proposes that there are two dimensions to consider: a causal dimension that considers the interplay between the learners’ characteristics and task, and an assessment dimension that focuses on measurable aspects of mental load, mental effort, and performance [15]. Several articles were dedicated to employing cognitive theory in online learning with the goal of enhancing students’ learning effectiveness [29–32]. Nonetheless, limited research has been conducted to examine and compare the cognitive load experienced by students in synchronous and asynchronous online modules. In this study, we revealed that the cognitive load in the synchronous online method is significantly lower than that in the asynchronous method, especially the ML. However, the effect sizes were small. Synchronous meetings could have provided instructors with the chance to support students in developing self-regulatory behaviors [33]. This includes activities like collectively navigating the learning management system and online textbook, addressing technical issues, and assisting students with time management. This finding aligns with a prior study, revealing that students in predominantly synchronous settings reported increased support for their basic psychological needs, particularly in competence and relatedness [34]. However, engaging in multiple tasks simultaneously during a synchronous virtual class, such as listening to the teacher, viewing presentation slides, processing new information, typing responses in the chat box, and reading comments, can increase cognitive load [35]. On the other hand, when students watch recordings of classes, they have more control over the learning process [36]. They can pause and rewind the recording, allowing them more time to process the information and thereby reducing cognitive load. Considering both the favorable and unfavorable research outcomes, further investigation into this finding is warranted in the future.

Numerous studies have researched the influence of video speed on learning efficiency in asynchronous education. Platforms like YouTube offer users the flexibility to modify video playback speed, facilitating an experience that can be twice as swift. In addition to entertainment content, students frequently adjust the speed of asynchronous lecture videos. A survey conducted among 123 undergraduate students at the University of California, Los Angeles (UCLA), revealed that 85% of participants watch lecture videos at speeds surpassing the standard rate [37]. Earlier investigations on the impact of video speed on learning outcomes have yielded inconclusive results. Studies by Lang et al. (2020), Nagahama & Morita (2017), Wilson et al. (2018), and Murphy et al. (2022) provide evidence suggesting that increasing video speed either maintains or enhances comprehension [37–40]. Conversely, works such as those by Song et al. (2018), and Vemuri et al. (2004) propose that heightened speed may impede comprehension [41, 42]. In examining the impact of adjusting playback speeds on medical education, there were 2 articles that provided valuable insights [43, 44]. Both studies present evidence suggesting that varying playback speeds do not significantly affect memory retention in medical students. However, in this study, data regarding video speed and learning outcomes were not collected. Future research will gather relevant data to analyze the correlation between video speed, students’ learning performance, self-efficacy, and cognitive load.

Regarding students’ satisfaction, the satisfaction with synchronous and asynchronous online methods is similar. The present finding aligns with previous meta-analyses that indicated a slightly higher level of satisfaction in synchronous environments, such as webinars, compared to asynchronous online instruction [45, 46]. It suggests that neither asynchronous nor synchronous online education significantly influences learning or teacher satisfaction.

Certain limitations of the current study deserve mention. Firstly, the data was derived from a single Taiwan university, and the participants were medical students. The results would only be generalized to a limited extent. However, universities in Taiwan are equipped similarly with the basic infrastructure. Therefore, we presume that the findings can be applied elsewhere, particularly within the context of Taiwan. Additionally, the study’s design, allowing students to choose their preferred method of accessing online lectures, aimed to fulfill students’ autonomy needs but introduced potential selection bias. Nevertheless, adhering to the self-determination theory, meeting the need for autonomy is anticipated to enhance intrinsic motivation (IM), positively correlating with academic performance [47]. Another limitation pertains to the inability to ascertain the individual duration students spent on asynchronous online videos or whether they completed entire lectures. Access to video content was not restricted, allowing students attending the synchronous lecture to view asynchronous content; however, no overlapping data in the analysis of the two student groups was observed. Furthermore, the identical quizzes administered in both the pretest and posttest, with differing quizzes in the retention test, pose a limitation, impeding direct comparability. Furthermore, the measurement’s validity is constrained by only five quizzes in each test. The study acknowledges the challenge of potential cheating in the pretest and posttest, although it did not impact students’ grades, but cheating was prevented in the on-site retention test. Lastly, the study’s one-shot design featuring a single lecture poses a significant challenge, constraining its correlational scope and hindering the establishment of causal relationships, despite theoretical assumptions that might imply otherwise. These limitations collectively indicate the need for future research strategies, such as conducting randomized studies or encompassing additional preclinical lectures.”

## Conclusion

The objective of this study was to investigate students’ academic performance, self-efficacy, and cognitive load between synchronous and asynchronous online learning formats. The results demonstrated no significant difference in improving learning performance and self-efficacy between the two modalities. However, a notable finding was that the cognitive load was significantly lower in the synchronous module with small effect sizes. These findings underscore the importance for educators in health professions to take into account students’ cognitive load during the development of online curricula.

### Electronic supplementary material

Below is the link to the electronic supplementary material.

Supplementary Material 1

Supplementary Material 2

Supplementary Material 3

Supplementary Material 4

Supplementary Material 5

Supplementary Material 6

Supplementary Material 7

## Data Availability

The datasets used and/or analyzed during the current study are available from the corresponding author on reasonable request.

## References

[1] Kang H, Zhang J, Kang J. Analysis of online education reviews of universities using NLP techniques and statistical methods. Wirel Commun Mob Com. 2022.

[2] Garcia MB. Socioeconomic inclusion during an era of online education. IGI Global; 2022.

[3] Hodges CB, Moore S, Lockee BB, Trust T, Bond MA. The difference between emergency remote teaching and online learning. 2020.

[4] Persada SF, Prasetyo YT, Suryananda XV, Apriyansyah B, Ong AK, Nadlifatin R, Setiyati EA, Putra RAK, Purnomo A, Triangga B (2022). How the Education Industries React to Synchronous and Asynchronous Learning in COVID-19: Multigroup Analysis insights for Future Online Education. Sustainability.

[5] Murphy E, Rodríguez-Manzanares MA, Barbour M (2011). Asynchronous and synchronous online teaching: perspectives of Canadian high school distance education teachers. Br J Edu Technol.

[6] Bernard RM, Abrami PC, Lou Y, Borokhovski E, Wade A, Wozney L, Wallet PA, Fiset M, Huang B (2004). How does distance education compare with classroom instruction? A meta-analysis of the empirical literature. Rev Educ Res.

[7] Kotrikadze EV, Zharkova LI (2021). Advantages and disadvantages of distance learning in universities. Propósitos Y Representaciones.

[8] Dinh LP, Nguyen TT. Convenient and comfortable, yet limited in many ways: advantages and disadvantages of online learning during the COVID-19 pandemic from perspectives of social work students in Vietnam. Asia Pac J Social Work Dev. 2022:1–9.

[9] Dziuban C, Moskal P, Thompson J, Kramer L, DeCantis G, Hermsdorfer A (2015). Student satisfaction with online learning: is it a psychological contract?. Online Learn.

[10] Bandura A (1977). Self-efficacy: toward a unifying theory of behavioral change. Psychol Rev.

[11] Locke EA (1997). Self-efficacy: the exercise of control. Pers Psychol.

[12] Multon KD, Brown SD, Lent RW (1991). Relation of self-efficacy beliefs to academic outcomes: a meta-analytic investigation. J Couns Psychol.

[13] Swan K (2004). Learning online: a review of current research on issues of interface, teaching presence and learner characteristics. Elem Qual Online Education: into Mainstream.

[14] Young JQ, Van Merrienboer J, Durning S, Ten Cate O (2014). Cognitive load theory: implications for medical education: AMEE Guide 86. Med Teach.

[15] Paas FG, Van Merriënboer JJ (1994). Instructional control of cognitive load in the training of complex cognitive tasks. Educational Psychol Rev.

[16] Paas F, Tuovinen JE, Tabbers H, Van Gerven PW (2003). Cognitive load measurement as a means to advance cognitive load theory. Educational Psychol.

[17] Sweller J, Ayres P, Kalyuga S. Cognitive load theory. Springer New York; 2011.

[18] Kirschner PA. Cognitive load theory: implications of cognitive load theory on the design of learning. Elsevier; 2002;12:1–10.

[19] Nieuwoudt JE (2020). Investigating synchronous and asynchronous class attendance as predictors of academic success in online education. Australasian J Educational Technol.

[20] Hung CT, Fang SA, Liu FC, Hsu CH, Yu TY, Wang WM (2022). Applying the Student Response System in the Online Dermatologic Video Curriculum on Medical Students’ Interaction and Learning outcomes during the COVID-19 pandemic. Indian J Dermatol.

[21] Pintrich PR. A manual for the use of the motivated strategies for learning questionnaire (MSLQ). 1991.

[22] Hwang G-J, Yang L-H, Wang S-Y (2013). A concept map-embedded educational computer game for improving students’ learning performance in natural science courses. Comput Educ.

[23] Allen M, Mabry E, Mattrey M, Bourhis J, Titsworth S, Burrell N (2004). Evaluating the effectiveness of distance learning: a comparison using meta-analysis. J Communication.

[24] Bandura A (1986). The explanatory and predictive scope of self-efficacy theory. J Soc Clin Psychol.

[25] Bulfone G, Fida R, Ghezzi V, Macale L, Sili A, Alvaro R, Palese A (2016). Nursing student self-efficacy in psychomotor skills: findings from a validation, longitudinal, and correlational study. Nurse Educ.

[26] Ober TM, Brodsky JE, Lodhi A, Brooks PJ. How did introductory psychology students experience the transition to remote online instruction amid the COVID-19 outbreak in New York City? Scholarsh Teach Learn Psychol. 2021.

[27] Gravelle CD, Roberts R, Che ES, Lodhi AK, Zapparrata NM, Ober TM, Brodsky JE, Brooks PJ. Online course formats and student self-efficacy in academic skills predict persistence in introductory psychology. Scholarsh Teach Learn Psychol. 2023.

[28] Shea P, Bidjerano T (2010). Learning presence: towards a theory of self-efficacy, self-regulation, and the development of a communities of inquiry in online and blended learning environments. Comput Educ.

[29] Chen C-M, Wu C-H (2015). Effects of different video lecture types on sustained attention, emotion, cognitive load, and learning performance. Comput Educ.

[30] Wang C, Fang T, Gu Y (2020). Learning performance and behavioral patterns of online collaborative learning: impact of cognitive load and affordances of different multimedia. Comput Educ.

[31] Salem MA, Sobaih AEE. ADIDAS: an examined approach for enhancing cognitive load and attitudes towards synchronous digital learning amid and post COVID-19 pandemic. Int J Environ Res Public Health. 2022;19(24). 10.3390/ijerph192416972 PMC9779740 36554852

[32] Zhang Y, Yang J, Wen ZE (2023). Learners with low Working Memory Capacity Benefit more from the Presence of an instructor’s Face in Video lectures. J Intell.

[33] Borup J, Graham CR, West RE, Archambault L, Spring KJ (2020). Academic communities of engagement: an expansive lens for examining support structures in blended and online learning. Education Tech Research Dev.

[34] Fabriz S, Mendzheritskaya J, Stehle S (2021). Impact of synchronous and asynchronous settings of online teaching and learning in higher education on students’ learning experience during COVID-19. Front Psychol.

[35] Chandler P, Sweller J (1991). Cognitive load theory and the format of instruction. Cognition Instruction.

[36] Li C-S, Irby B (2008). An overview of online education: attractiveness, benefits, challenges, concerns and recommendations. Coll Student J.

[37] Murphy DH, Hoover KM, Agadzhanyan K, Kuehn JC, Castel AD (2022). Learning in double time: the effect of lecture video speed on immediate and delayed comprehension. Appl Cogn Psychol.

[38] Lang D, Chen G, Mirzaei K, Paepcke A. Is faster better? A study of video playback speed. In: *Proceedings of the tenth international conference on learning analytics & knowledge: 2020*; 2020:260–269.

[39] Nagahama T, Morita Y. Effect analysis of playback speed for lecture video including instructor images. Int J Educational Media Technol 2017, 11(1).

[40] Wilson KE, Martin L, Smilek D, Risko EF (2018). The benefits and costs of speed watching video lectures. Scholarsh Teach Learn Psychol.

[41] Song K, Chakraborty A, Dawson M, Dugan A, Adkins B, Doty C (2018). Does the podcast video playback speed affect comprehension for novel curriculum delivery? A randomized trial. Western J Emerg Med.

[42] Vemuri S, DeCamp P, Bender W, Schmandt C. Improving speech playback using time-compression and speech recognition. In: *Proceedings of the SIGCHI conference on Human factors in computing systems: 2004*; 2004:295–302.

[43] Merhavy ZI, Bassett L, Melchiorre M, Hall MP (2023). The impact of lecture playback speeds on concentration and memory. BMC Med Educ.

[44] Kiyak YS, Budakoglu II, Masters K, Coskun O (2023). The effect of watching lecture videos at 2x speed on memory retention performance of medical students: an experimental study. Med Teach.

[45] Ebner C, Gegenfurtner A. Learning and satisfaction in webinar, online, and face-to-face instruction: a meta-analysis. Frontiers in education: 2019. Frontiers Media SA; 2019:92.

[46] Xu T, Xue L. Satisfaction with online education among students, faculty, and parents before and after the COVID-19 outbreak: evidence from a meta-analysis. Front Psychol. 2023;14. 10.3389/fpsyg.2023.1128034 PMC9968937 36860782

[47] Ten Cate TJ, Kusurkar RA, Williams GC (2011). How self-determination theory can assist our understanding of the teaching and learning processes in medical education. AMEE Guide 59 Med Teach.

