# Supplementary material for: The evaluation of synchronous and asynchronous online learning: student experience, learning outcomes, and cognitive load
Source: BMC Med Educ. 2024 Mar 22;24:326. doi: 10.1186/s12909-024-05311-7 (PMC10960437; doi:10.1186/s12909-024-05311-7)
Supplement: Supplementary file 2 — Supplementary Material 2 [file 12909_2024_5311_MOESM2_ESM.doc]

Table S2. Comparison of Self-Efficacy for Learning and Performance in pre-/post- Asynchronous module

|  | | Pre-lecture | Post-lecture | *p* value |
| --- | --- | --- | --- | --- |
| Total | | 3.88 | 4.26 | < .0001 |
| 1 | I believe I will receive an excellent grade in this class | 3.87 | 4.22 | < .0001 |
| 2 | I'm certain I can understand the most difficult material presented in the readings for this course. | 3.78 | 4.18 | < .0001 |
| 3 | I'm confident I can understand the basic concepts taught in this course. | 3.97 | 4.38 | < .0001 |
| 4 | I'm confident I can understand the most complex material presented by the instructor in this course. | 3.79 | 4.26 | < .0001 |
| 5 | I'm confident I can do an excellent job on the assignments and tests in this course. | 3.87 | 4.15 | .0007 |
| 6 | I expect to do well in this class. | 4.02 | 4.34 | < .0001 |
| 7 | I'm certain I can master the skills being taught in this class. | 3.8 | 4.21 | < .0001 |
| 8 | Considering the difficulty of this course, the teacher, and my skills, I think I will do well in this class. | 3.97 | 4.35 | < .0001 |
