# Supplementary material for: The evaluation of synchronous and asynchronous online learning: student experience, learning outcomes, and cognitive load
Source: BMC Med Educ. 2024 Mar 22;24:326. doi: 10.1186/s12909-024-05311-7 (PMC10960437; doi:10.1186/s12909-024-05311-7)
Supplement: Supplementary file 3 — Supplementary Material 3 [file 12909_2024_5311_MOESM3_ESM.doc]

Table S3. Comparison of Self-Efficacy for Learning and Performance in pre-lecture between Synchronous and Asynchronous modules

| Pre-lecture | | Synchronous | Asynchronous | *p* value |
| --- | --- | --- | --- | --- |
| 1 | I believe I will receive an excellent grade in this class | 3.89 | 3.87 | .9979 |
| 2 | I'm certain I can understand the most difficult material presented in the readings for this course. | 3.81 | 3.78 | .6814 |
| 3 | I'm confident I can understand the basic concepts taught in this course. | 4.07 | 3.97 | .2752 |
| 4 | I'm confident I can understand the most complex material presented by the instructor in this course. | 3.79 | 3.79 | .9637 |
| 5 | I'm confident I can do an excellent job on the assignments and tests in this course. | 3.81 | 3.87 | .4786 |
| 6 | I expect to do well in this class. | 4 | 4.02 | .8939 |
| 7 | I'm certain I can master the skills being taught in this class. | 3.87 | 3.8 | .4812 |
| 8 | Considering the difficulty of this course, the teacher, and my skills, I think I will do well in this class. | 4.01 | 3.97 | .6822 |
