# Supplementary material for: The evaluation of synchronous and asynchronous online learning: student experience, learning outcomes, and cognitive load
Source: BMC Med Educ. 2024 Mar 22;24:326. doi: 10.1186/s12909-024-05311-7 (PMC10960437; doi:10.1186/s12909-024-05311-7)
Supplement: Supplementary file 4 — Supplementary Material 4 [file 12909_2024_5311_MOESM4_ESM.doc]

Table S4. Comparison of Self-Efficacy for Learning and Performance in Post-lecture between Synchronous and Asynchronous modules

| Post-lecture | | Synchronous | Asynchronous | *p* value |
| --- | --- | --- | --- | --- |
| 1 | I believe I will receive an excellent grade in this class | 4.25 | 4.22 | .7876 |
| 2 | I'm certain I can understand the most difficult material presented in the readings for this course. | 4.23 | 4.18 | .8059 |
| 3 | I'm confident I can understand the basic concepts taught in this course. | 4.44 | 4.38 | .4826 |
| 4 | I'm confident I can understand the most complex material presented by the instructor in this course. | 4.23 | 4.26 | .7271 |
| 5 | I'm confident I can do an excellent job on the assignments and tests in this course. | 4.23 | 4.15 | .6980 |
| 6 | I expect to do well in this class. | 4.31 | 4.34 | .8201 |
| 7 | I'm certain I can master the skills being taught in this class. | 4.21 | 4.21 | .9669 |
| 8 | Considering the difficulty of this course, the teacher, and my skills, I think I will do well in this class. | 4.37 | 4.35 | .7040 |
