# Supplementary material for: The evaluation of synchronous and asynchronous online learning: student experience, learning outcomes, and cognitive load
Source: BMC Med Educ. 2024 Mar 22;24:326. doi: 10.1186/s12909-024-05311-7 (PMC10960437; doi:10.1186/s12909-024-05311-7)
Supplement: Supplementary file 5 — Supplementary Material 5 [file 12909_2024_5311_MOESM5_ESM.doc]

Table S5. Comparison of cognitive load between Synchronous and Asynchronous modules

| Post-lecture | | Synchronous | Asynchronous | *p* value |
| --- | --- | --- | --- | --- |
| Cognitive load | | 2.53 | 2.84 | .0001 |
| **Mental load** | | 2.52 | 2.86 | .0005 |
| 1 | The learning content in this learning activity was difficult for me. | 2.7 | 3.06 | .1362 |
| 2 | I had to put a lot of effort into answering the questions in this learning activity. | 2.63 | 3.12 | .0380 |
| 3 | It was troublesome for me to answer the questions in this learning activity. | 2.47 | 2.79 | .1294 |
| 4 | I felt frustrated answering the questions in this learning activity. | 2.43 | 2.69 | .2103 |
| 5 | I did not have enough time to answer the questions in this learning activity. | 2.39 | 2.66 | .1358 |
| **Mental effort** | | 2.548 | 2.79 | .0662 |
| 6 | During the learning activity, the way of instruction or learning content presentation caused me a lot of mental effort. | 2.34 | 2.68 | .1462 |
| 7 | I need to put lots of effort into completing the learning tasks or achieving the learning objectives in this learning activity. | 2.87 | 3.07 | .3328 |
| 8 | The instructional way in the learning activity was difficult to follow and understand. | 2.43 | 2.62 | .5041 |
