# Supplementary material for: The evaluation of synchronous and asynchronous online learning: student experience, learning outcomes, and cognitive load
Source: BMC Med Educ. 2024 Mar 22;24:326. doi: 10.1186/s12909-024-05311-7 (PMC10960437; doi:10.1186/s12909-024-05311-7)
Supplement: Supplementary file 7 — Supplementary Material 7 [file 12909_2024_5311_MOESM7_ESM.docx]

|  |  | Strongly disagree | Disagree | Somewhat disagree | Neutral | Somewhat agree | Agree | Strongly agree |
| --- | --- | --- | --- | --- | --- | --- | --- | --- |
| 1 | The learning content in this learning activity was difficult for me. |  |  |  |  |  |  |  |
| 2 | I had to put a lot of effort into answering the questions in this learning activity. |  |  |  |  |  |  |  |
| 3 | It was troublesome for me to answer the questions in this learning activity. |  |  |  |  |  |  |  |
| 4 | I felt frustrated answering the questions in this learning activity. |  |  |  |  |  |  |  |
| 5 | I did not have enough time to answer the questions in this learning activity. |  |  |  |  |  |  |  |
| 6 | During the learning activity, the way of instruction or learning content presentation caused me a lot of mental effort. |  |  |  |  |  |  |  |
| 7 | I need to put lots of effort into completing the learning tasks or achieving the learning objectives in this learning activity. |  |  |  |  |  |  |  |
| 8 | The instructional way in the learning activity was difficult to follow and understand. |  |  |  |  |  |  |  |

Appendix 2. Questionnaire of cognitive load
